# Exploring the modulatory role of bovine lactoferrin on the microbiome and the immune response in healthy and Shiga toxin-producing *E. coli* challenged weaned piglets

**DOI:** 10.1186/s40104-023-00985-3

**Published:** 2024-03-06

**Authors:** Matthias Dierick, Ruben Ongena, Daisy Vanrompay, Bert Devriendt, Eric Cox

**Affiliations:** 1 Laboratory of Immunology, Department of Translational Physiology, Infectiology and Public Health, Faculty of Veterinary Medicine, Ghent University, Salisburylaan 133, 9820 Merelbeke, Belgium; 2 Laboratory for Immunology and Animal Biotechnology, Faculty of Bioscience Engineering, Ghent University, Coupure links 653, 9000 Ghent, Belgium

**Keywords:** *E. coli*, Immune modulation, Lactoferrin, Microbiome

## Abstract

**Background:**

Post-weaned piglets suffer from F18^+^ *Escherichia coli* (*E. coli*) infections resulting in post-weaning diarrhoea or oedema disease. Frequently used management strategies, including colistin and zinc oxide, have contributed to the emergence and spread of antimicrobial resistance. Novel antimicrobials capable of directly interacting with pathogens and modulating the host immune responses are being investigated. Lactoferrin has shown promising results against porcine enterotoxigenic *E. coli* strains, both in vitro and in vivo.

**Results:**

We investigated the influence of bovine lactoferrin (bLF) on the microbiome of healthy and infected weaned piglets. Additionally, we assessed whether bLF influenced the immune responses upon Shiga toxin-producing *E. coli* (STEC) infection. Therefore, 2 in vivo trials were conducted: a microbiome trial and a challenge infection trial, using an F18^+^ STEC strain. BLF did not affect the α- and β-diversity. However, bLF groups showed a higher relative abundance (RA) for the Actinobacteria phylum and the *Bifidobacterium* genus in the ileal mucosa. When analysing the immune response upon infection, the STEC group exhibited a significant increase in F18-specific IgG serum levels, whereas this response was absent in the bLF group.

**Conclusion:**

Taken together, the oral administration of bLF did not have a notable impact on the α- and β-diversity of the gut microbiome in weaned piglets. Nevertheless, it did increase the RA of the Actinobacteria phylum and *Bifidobacterium* genus, which have previously been shown to play an important role in maintaining gut homeostasis. Furthermore, bLF administration during STEC infection resulted in the absence of F18-specific serum IgG responses.

## Background

F18-fimbriated enterotoxigenic *Escherichia coli* (ETEC) and Shiga toxin-producing *E. coli* (STEC) are commonly associated with post-weaning diarrhoea (PWD) and oedema disease in piglets, respectively [1–3]. In Europe, F18-fimbriated ETEC strains are the second most prevalent *E. coli* strain isolated from piglets with PWD [4]. These strains typically produce enterotoxins, such as heat-labile enterotoxin (LT) or heat-stable enterotoxins (STa and STb). F18-fimbriated STEC strains produce Shiga toxin type 2e (Stx2e) and are the causative agent of oedema disease [1]. Both ETEC and STEC infections are controlled by the extensive use of antibiotics during the first two weeks after weaning, which may have contributed to the increased incidence of multi-drug resistant bacterial strains [5–8]. Consequently, there is an urgent need to reduce antibiotic usage, including the discontinuation of certain antibiotics, like colistin, which is considered a last-resort antibiotic in human medicine [9]. Several initiatives are boosting the development of alternatives strategies, such as the use of naturally occurring molecules, which can directly act on the pathogen and/or enhance host resistance [10, 11]. Lactoferrin (LF) is one of these molecules that has recently gained attention.

LF is a multifunctional iron-binding glycoprotein found in milk and other biological fluids, such as saliva, tears, nasal and bronchial secretions. The highest LF concentrations are found in colostrum and milk. For instance, the porcine colostrum contains porcine LF (pLF) at a concentration ranging from approximately 1.1–1.3 mg/mL, but this concentration sharply decreases during the initial week of lactation, reaching about 0.1 to 0.3 mg/mL [12]. However, a more recent study has shown that the concentration of pLF in porcine colostrum can be much higher, reaching concentration of about 8–10 mg/mL [13]. LF is part of the transferrin family and is composed of a single polypeptide chain with a molecular weight of 77 kDa [14, 15]. The protein consists of a C- and N-lobe separated by a short hinge region and has a variety of functions, like iron homeostasis, anti-inflammatory and antimicrobial activities [16–19]. Additionally, LF may also act as a mediator of the host immune system due to its ability to directly interact with microbe-associated molecular patterns (MAMPs), such as lipopolysaccharides (LPS). Furthermore, LF can act as a chemoattractant, enabling the recruitment of neutrophils and monocytes, and promotes differentiation and maturation of B- and T-cells [18, 20, 21]. A previous study in which milk replacer supplemented with bovine lactoferrin (bLF) was given to neonatal piglets, showed that immune cell populations in peripheral blood and ileal Peyer’s patches were unaffected. However, serum samples of piglets receiving the highest quantity of bLF (1,300 mg bLF/kg bodyweight/d) tended to have higher total serum IgG levels compared to the control, receiving 130 mg bLF/kg bodyweight/d [22]. Additionally, upon experimental infection of sheep with enterohemorrhagic *E. coli* O157:H7 (EHEC), bLF (1.5 g/12 h) enhanced the IgG serum response against the type 3 secretion system antigens EspA and EspB [23].

On the other hand, LF has also been shown to affect the microbiome in mice, human infants and suckling piglets [24–26]. For example, the amount of healthy microbes, such as Lactobacilli and Bifidobacteria, was positively correlated with the concentration of LF in the faeces of human infants [27]. Pre-weaned piglets exhibited comparable findings, with increased levels of *Bifidobacterium* spp. and *Lactobacillus* spp., along with decreased levels of pathogen-associated microbes, such as *Salmonella* [28]. More recently, oral administration of bLF in suckling piglets resulted in a significant increase in the bacterial richness estimators ACE and Chao1 [26]. However, the impact of orally administering LF on the gut microbiome of weaned piglets has not been thoroughly investigated.

Given the known antimicrobial activities of LF, the oral administration of LF could have adverse effects on the gut microbiome. Therefore, we wanted to assess the potential impact of dietary bLF on the microbiome of healthy and STEC infected weaned piglets. More specifically, we evaluated the impact of orally administered LF on the gut microbiome, not only in faecal matter but also in intestinal content. In addition, our prior research established LF's antibacterial properties against porcine ETEC/STEC strains, such as the degradation of virulence factors linked to porcine ETEC/STEC strains, and its mitigation of ETEC-induced fluid secretion [29, 30]. To substantiate these findings and examine whether LF can enhance host immune responses against STEC, we conducted a challenge infection experiment. This enabled us to investigate the impact of dietary bLF on both local and systemic immune responses following an STEC infection.

## Materials and methods

### Animals

**Fig. 1:**
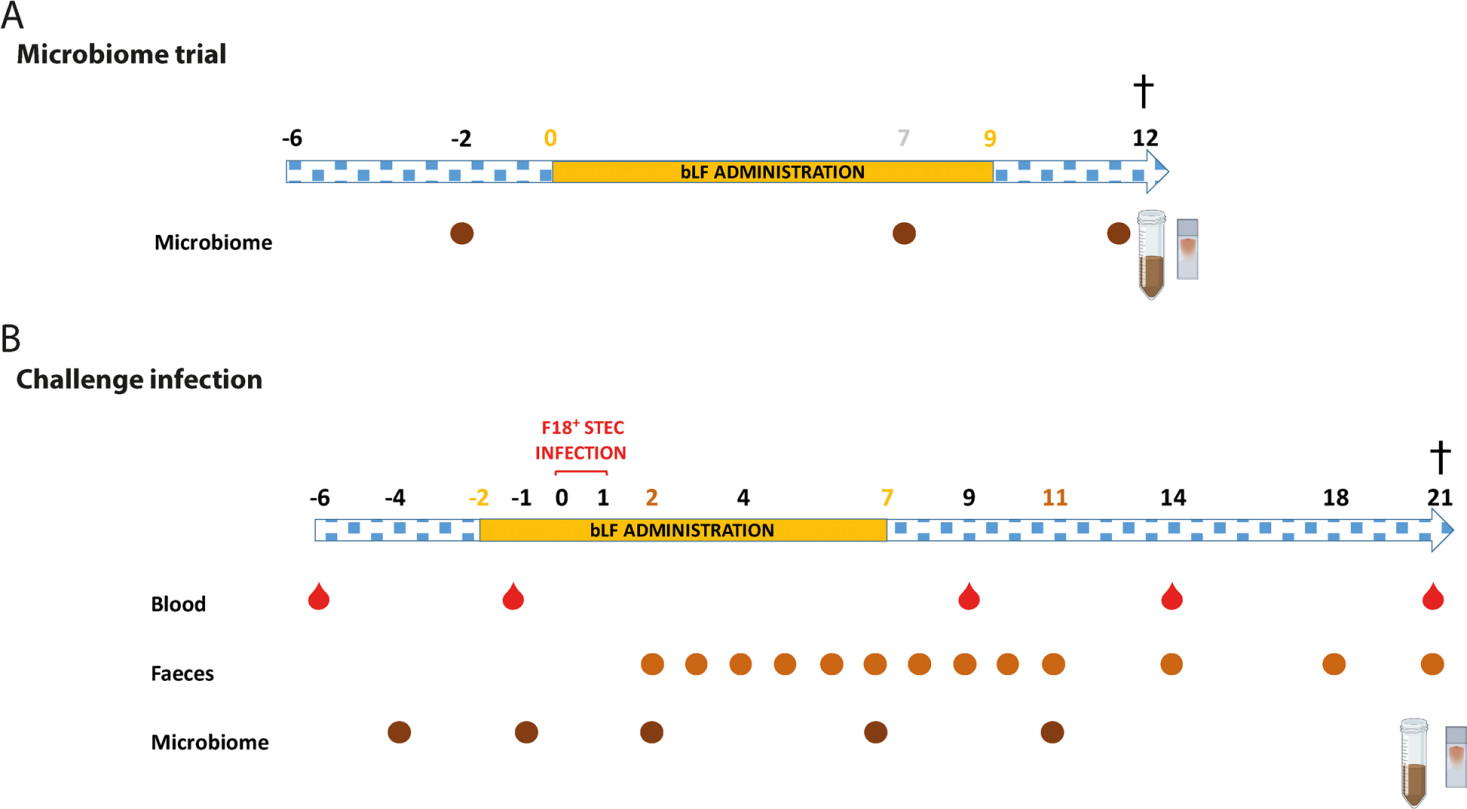
Timeline of the bLF microbiome trial and F18^+^ STEC challenge in vivo. In both trials, bovine lactoferrin (bLF) was administered orally twice a day (for a total of 500 mg/d) for 10 consecutive days. To study the gut microbiome different samples were taken: faecal swabs were collected during the experiments, as indicated in the timeline, and mucosal scrapings and intestinal content of both ileum and colon were sampled upon euthanasia, indicated by †. The challenge infection with an F18^+^ STEC strain (F107/86) was carried out on D0 and D1. Blood and faeces were collected on different timepoint to determine effect of bLF on F18-specific immune responses and faecal shedding. bLF: bovine lactoferrin, STEC: Shiga toxin-producing *E. coli*

Two in vivo experiments, a microbiome trial (Fig. 1A) with 14 (6 female, 8 male) and an infection trial (Fig. 1B) with 10 (1 female, 9 male), conventionally reared piglets (Landrace × Pietrain), were performed. These piglets were selected to be F18-seronegative, as determined by ELISA, and F18-receptor positive, using FUT1 genotyping [31, 32]. In the infection trial (*n* = 10), 10 piglets were randomly assigned to the STEC control (*n* = 5; 4 males and 1 female) or STEC + bLF (*n* = 5; 5 males) group. For the microbiome trial (*n* = 14), piglets from different sows were distributed evenly over the two experimental groups to minimize potential biases related to genetic background. Consequently, both the control and bLF groups comprised 7 animals each (*n* = 7), consisting of 3 females and 4 males in each experimental group. All piglets were weaned at an age of 4 weeks and subsequently transported to our facilities where they were housed in isolation units and allowed to acclimatize for 1 week.

The challenge trial (*n* = 10) was used to assess the effect of bLF (derived from bovine milk, with a purity of 92% and 16% iron saturation; Ingredia Nutritional, Arras, France) on the excretion of and immune response against an *E. coli* infection. Additionally, the effect of bLF on the microbiome (*n* = 14) in the presence or absence of a challenge infection with an F18^+^ *E. coli* strain was investigated. These experiments were reviewed and approved by the Ethical Committee of the Faculty of Veterinary Medicine at Ghent University, in accordance with the Belgian law on animal experimentation (EC2019/085 and EC2021/080).

### Oral administration of bovine lactoferrin

In the microbiome in vivo experiment (Fig. 1A), animals were again randomly assigned to two groups: (i) a control group (*n* = 7) and an experimental group (*n* = 7), receiving either PBS or bLF, respectively. In the in vivo challenge experiment (Fig. 1B), animals were randomly assigned to 2 groups: (i) an infection control group (*n* = 5) and (ii) an infection group receiving bLF (*n* = 5). BLF (500 mg/d; dissolved in filter-sterile PBS) was orally administered through drenching, using a syringe without a needle. This administration was divided into two daily administrations (at 8:00 and 17:00 h), each consisting of 250 mg bLF in 10 mL PBS, and this over a period of 10 consecutive days. No gastric pH neutralization was conducted prior to the oral administration of bLF.

### Oral challenge with F18^+^*E. coli*

BLF was administered orally to the piglets from 2 d prior until 7 d post challenge infection with the STEC strain F107/86 (F18ab^+^, O139:K12:H1, Stx2e^+^) (Fig. 1). Three hours prior to the challenge, piglets were deprived from food and water. All 10 pigs were challenged on two consecutive days (D0 and D1) with 10^11^ CFU F18ab^+^ STEC (F107/86) in 10 mL PBS after neutralizing the gastric pH by intragastric administration of 60 mL NaHCO_3_ (1.4% (w/v)) in distilled water [31].

Faecal samples were taken daily (D1–10) and additionally on D14, D18 and D21 post infection to monitor the F18^+^ STEC faecal excretion. Hereto, 100 μL of 10-fold dilutions of faeces in PBS, starting from a 1% (w/v) suspension, were plated onto blood agar plates, supplemented with 1 mg/mL streptomycin sulphate salt (Sigma, St Louis, MO, USA). After overnight incubation, F18^+^ STEC was identified using dot blotting and detection with an in-house monoclonal F18-specific antibody (IMM02) and anti-mouse-IgG-HRP (Dako, Glostrup, Denmark) [33]. Binding of the secondary antibody was visualized using a 3-amino-9-ethylcarbazole (AEC) solution.

Furthermore, blood was drawn from the jugular vein at D−6, D−1 and D9, D14 and D21 post infection to analyse serum antibody responses via ELISA and to assess the presence of antigen-specific IgA^+^ antibody secreting cells (ASC) in the peripheral blood mononuclear cell (PBMC) population using ELISpot [34]. At D21 post infection, animals were euthanized by intravenous injection with sodium-pentobarbital (Kela Health, Hoogstraten, Belgium) and upon exsanguination intestinal tissues were collected for the isolation of mononuclear cells (MCs) to quantify the number of F18-specific IgA^+^ ASCs in these tissues via ELISpot. Furthermore, intestinal villi were scraped from the jejunum and the F18 fimbriae receptor status was verified using an in vitro villus adhesion test, as previously described [35].

### F18-specific serum antibody ELISA

Serum was collected from blood, inactivated at 56 °C for 30 min and subsequently treated with kaolin [36]. A 96-well microtiter plate (Nunc, Maxisorp, Life Technologies, Merelbeke, Belgium) was coated with 2 μg/mL F18 fimbriae (in PBS) incubated for 2 h at 37 °C and subsequently blocked (PBS + 3% BSA + 0.2% Tween 80) overnight at 4 °C. The plates were then incubated with the sera of the pigs for 1 h at 37 °C (diluted 1/15) and with anti-pig IgA/IgG HRP (Bethyl Laboratories, Montgomery, TX, USA) for 1 h at 37 °C in dilution buffer (PBS + 3% BSA + 0.2% Tween 20). The reaction was visualized using 2,2'-azino-bis(3-ethylbenzothiazoline-6-sulfonic acid) (ABTS) (Roche, Mannheim, Germany) and measured spectrophotometrically (OD_405nm_) using a SPECTRA Fluor ELISA platereader (TECAN, Mannedorf, Switzerland).

### Enzyme-linked immunosorbent spot (ELISpot) to detect F18-specific antibody-secreting cells

MCs were isolated from blood (PBMC), mesenteric lymph nodes (MLN), jejunal Peyer’s patches (JPP), jejunal lamina propria (JLP), ileal Peyer’s patches (IPP) and ileal lamina propria (ILP) and processed as described [31, 37]. The MCs were isolated by density gradient centrifugation on Lymphoprep (Alere Technologies, Oslo, Norway) for 25 min, 800 × *g* at 18 °C and resuspended in CTL-Test™ B-medium (Cellular Technology Limited, Cleveland, OH, USA). MultiScreen filter plates (96-well format, MAIPA4510, Millipore, Darmstadt, Germany) were activated with 70% ethanol for 30 s, washed twice with ultrapure (UP) water and coated overnight at 4 °C with 15 μg/mL F18 fimbriae. Upon washing, the plates were incubated for 2 h at 37 °C with CTL-test B medium. PBMCs and MCs from MLN and other tissues (1 × 10^6^ cells/well) were added to the wells (5 × 10^5^ cells/well) and incubated for 18 h at 37 °C, 5% CO_2_ in a humidified atmosphere. Cells were then removed by intensive washing with PBS containing 0.1% Tween 20. Upon washing, biotin-conjugated IgA (1/10,000; Bethyl; A100-102B) was added in assay buffer (PBS + 0.1% Tween 20 + 0.1% BSA), incubated for 2 h at room temperature and subsequently incubated with streptavidin-HRP (1/1,000 Mabtech, Nacka Strand, Sweden; 3310-9-1000). Detection was performed by adding 3,3′,5,5′-Tetramethylbenzidine (TMB) substrate for membranes (Sigma, St Louis, MO, USA) and counting of the spots using an Immunospot reader (Cellular Technology Ltd., Cleveland, OH, USA).

### Analysis of the gut microbiome

During the F18^+^ STEC challenge study, faecal swabs were taken at D−4 , D−1, D4, D7 and D11. In the microbiome trial, faecal swabs were taken at D−2, D7 and D12 (Fig. 1). Upon euthanasia, mucosal scrapings were taken at the ileal sites with and without Peyer’s patches and at the colonic mucosa (caudal site). Mucosal scrapings were collected by gently scraping along the mucosal surface with a microscopy glass slide and stored at −80 °C. Furthermore, intestinal content of both ileum and colon was also collected at this time and stored at −80 °C.

Next, samples were sent out to Eurofins Genomics (Ebersberg, Germany) where DNA isolation, followed by next-generation amplicon sequencing and microbiome profiling was performed. After DNA isolation, the V3–V4 region of the bacterial 16S rRNA gene was amplified and sequenced using an Illumina MiSeq to identify bacterial operational taxonomic units (OTUs) following the standard procedure ‘InView—Microbiome Profiling 3.0 with MiSeq’. Sequences were demultiplexed, the primers were clipped, forward and reverse reads were merged and merged reads were quality filtered. As a first step of the microbiome analysis, reads with ambiguous bases (‘N’) were removed. Chimeric reads were identified and removed based on the de novo algorithm of UCHIME as implemented in the VSEARCH package [38, 39]. The remaining set of high-quality reads was processed using minimum entropy decomposition (MED) to position marker gene data sets into OTUs [40, 41]. Furthermore, the MED procedure identifies and filters random ‘noise’ in the data set, i.e., sequences with a very low abundance (< 0.02% of the average sample size). To assign taxonomic information to each OTU, DC-MEGABLAST alignments of cluster representative sequences to the sequence database were performed. The most specific taxonomic assignment for each OTU was then transferred from the set of best-matching reference sequences (lowest common taxonomic unit of all best hits). Hereby, a sequence identity of 70% across at least 80% of the representative sequence was a minimal requirement for considering reference sequences. Further processing of OTUs and taxonomic assignments was performed using the QIIME software package (version 1.9.1, http://qiime.org/) [42]. Abundances of bacterial taxonomic units were normalized using lineage-specific copy numbers of the relevant marker genes to improve estimates [43]. The richness and diversity was assessed on OTU level, based on ACE, Chao1, Shannon, and Simpson indices, and principal coordinates analysis (PCoA) was performed, using Bray-Curtis similarity clustering analysis. These analysis were performed using the phyloseq package (version 1.42.0) and vegan package (version 2.6.4) in RStudio (version 2022.12.0+353) [44, 45].

### Data analysis

Statistical analysis of data and design of figures were performed using Rstudio (version 2022.12.0+353) and GraphPad Prism 8 (GraphPad Software, San Diego, CA, USA). Alpha diversity and relative abundance of taxa were analysed using the Mann-Whitney U test. To assess variations in beta diversity among sample groups, a permutational multivariate analysis of variance (PERMANOVA) was performed. The adonis2 function from the R package vegan 2.6.4 was used for this analysis, with 999 permutations. The faecal excretion and serum immune response were analysed using a two way-ANOVA with a correction for multiple comparisons, performed by controlling the false discovery rate, using GraphPad Prism 8. The normality assumption was evaluated using the Shapiro-Wilk test, all *P*-values were found to be greater than 0.05. To assess the effect of bLF on F18-specific IgA^+^ ASC, a non-parametric Mann-Whitney U analysis was performed using GraphPad 8.

## Results

### Effect of lactoferrin on the gut microbiome of weaned piglets

Based on previous evidence indicating that bLF administration increased bacterial richness estimators and induced changes in microbiota composition in suckling piglets, we wanted to investigate whether similar effects could be observed in weaned piglets [26]. In this study, a total of 16.8 million V3–V4 16S rRNA sequence reads, from 224 samples with an average number of 75,281 sequence reads, were obtained and used in subsequent analyses. These samples included faecal swabs, mucosal scrapings from the ileal site and the colonic mucosa, and intestinal content from both the ileum and colon, from STEC challenged and unchallenged piglets (Fig. 1).

**Fig. 2:**
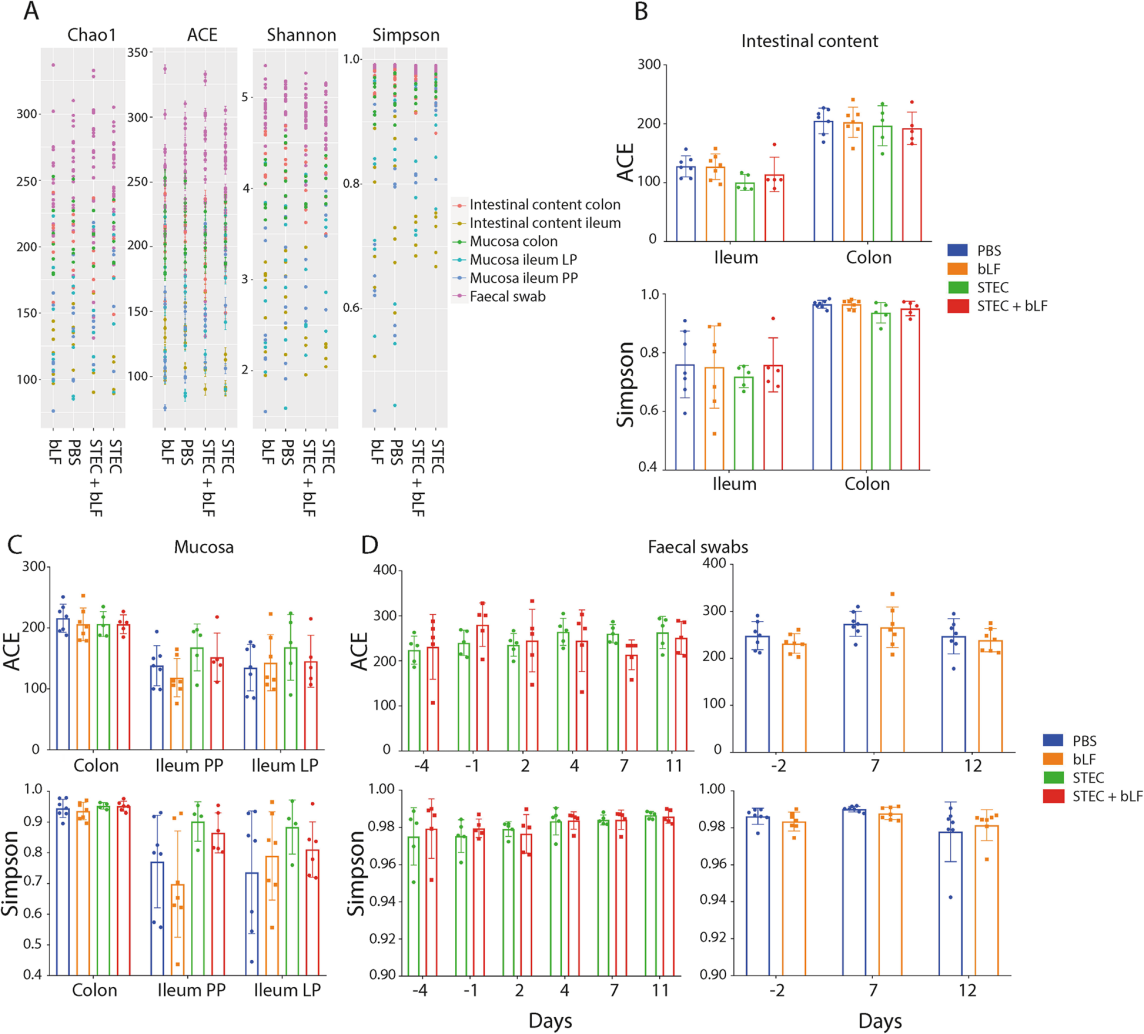
Effect of bLF administration on the microbiome richness and diversity in post-weaned piglets. **A** Chao1, ACE, Shannon and Simpson of all samples, **B–D** ACE and Simpson of faecal content (**B**), mucosal scraping (**C**), and faecal swabs (**D**). Values represented as mean ± SD; *n* = 7 (PBS and bLF) and *n* = 5 (STEC and STEC + bLF). bLF: bovine lactoferrin, LP: lamina propria, PP: Peyer’s patches

**Fig. 3:**
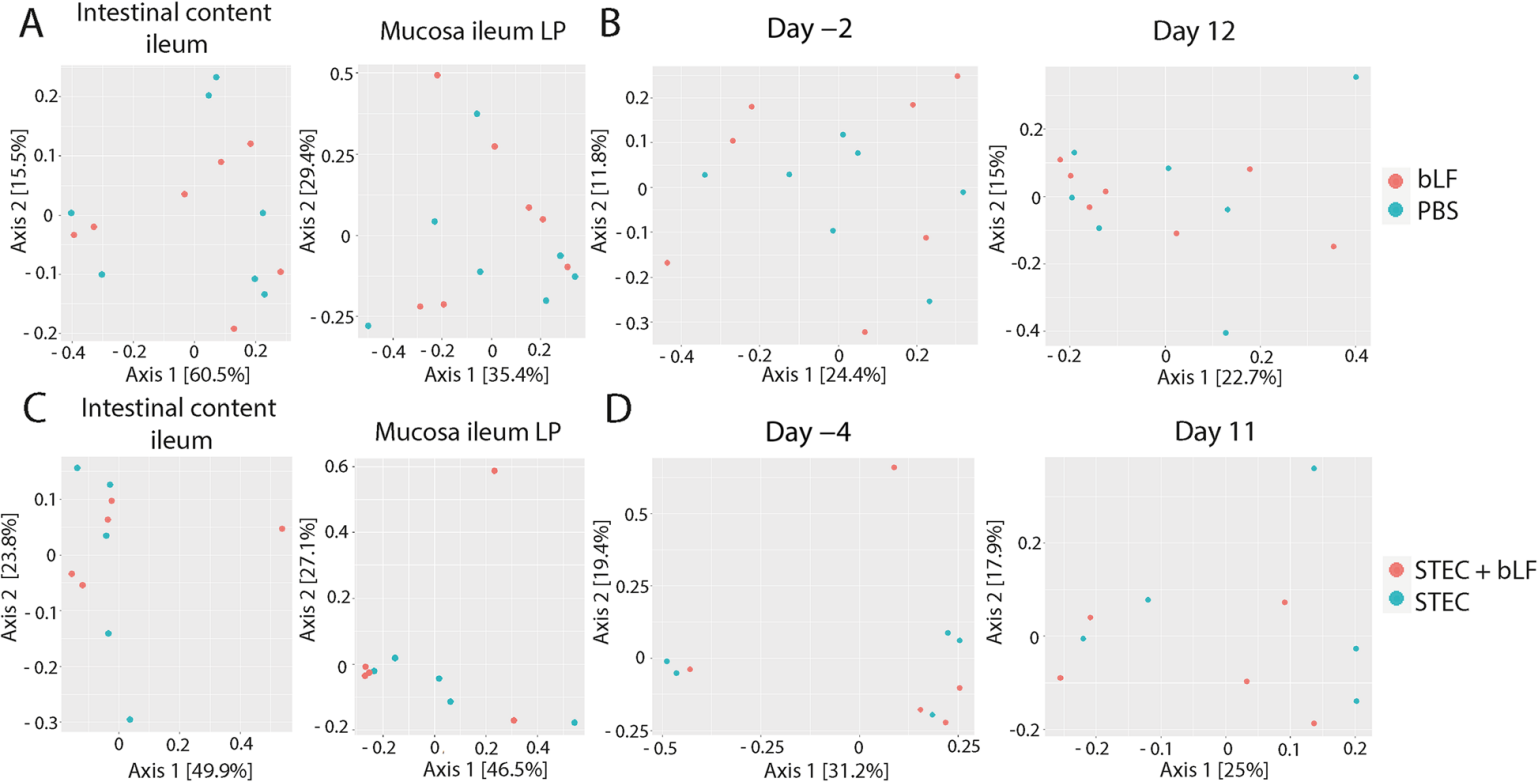
Principal coordinate analysis (PCoA) of bLF administration on the microbiome of post-weaned piglets. **A** and **B** PCoA of the microbiome trial experiment (*n* = 7), **C **and **D** PCoA of the STEC challenge trial (*n* = 5). **A** and **C** Faecal content ileum (left) and mucosal scraping ileum LP (right), **B **and **D** Faecal swabs. Data was analysed by PCoA analysis using the Bray-Curtis distance. bLF: bovine lactoferrin, LP: lamina propria, STEC: Shiga toxin-producing *E. coli*

To investigate the impact of bLF on the gut microbiota of weaned piglets, we conducted analyses to assess the bacterial richness and the microbial composition. Fig. 2 A shows the bacterial richness estimators (ACE and Chao1 indices) and diversity indices (Shannon and Simpson indices) for each sample. No significant effect of LF was observed on these bacterial richness and diversity indices (Fig. 2B–D). In order to determine whether the microbial composition differed between pigs that received bLF and those without bLF, a PCoA was performed. This analysis revealed that bLF did not alter the composition of the microbial community (Fig. 3).

**Fig. 4:**
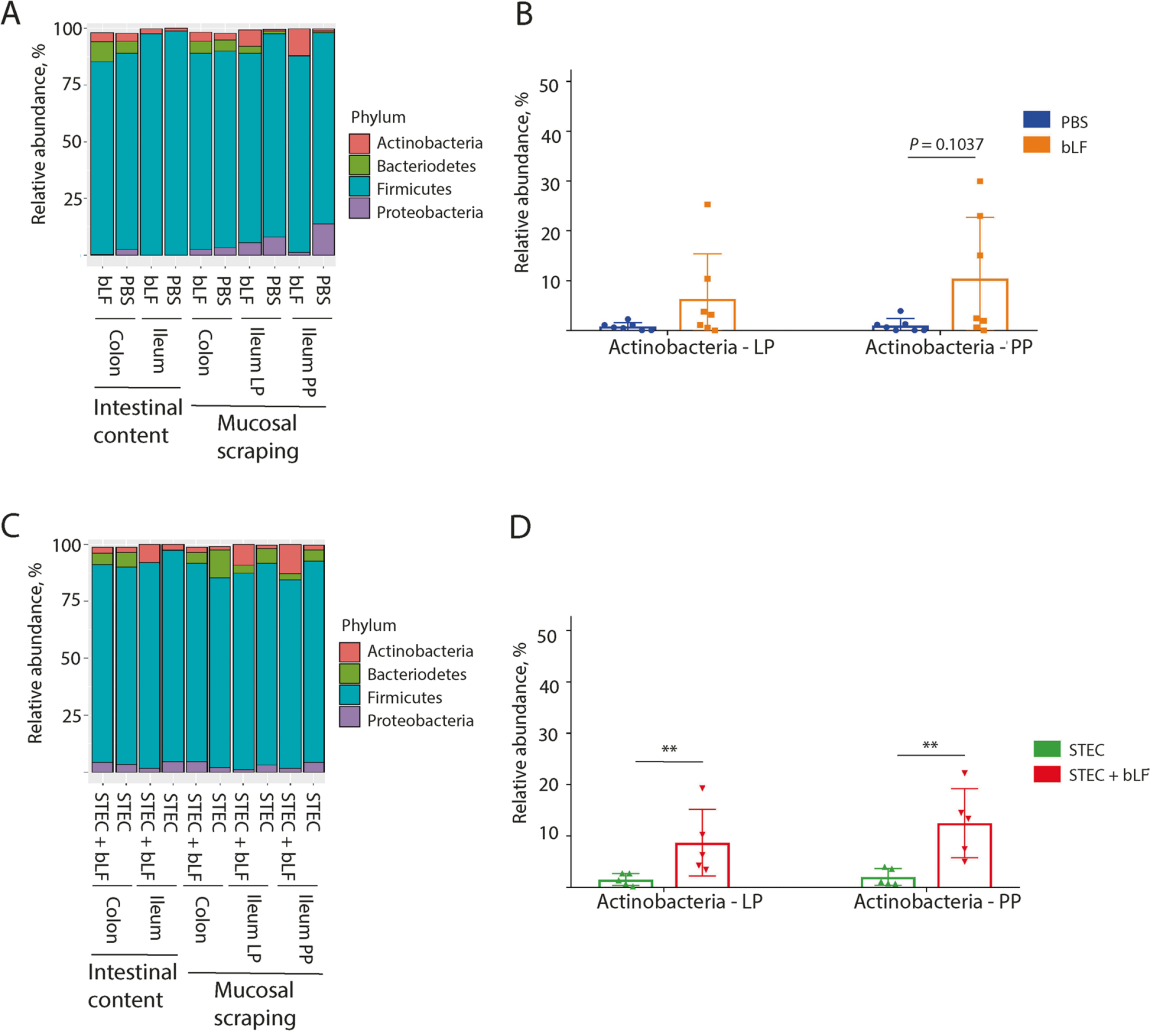
Effect of LF administration on the relative abundance of bacterial phyla. **A** and **C** Stacked bar chart of the 4 most abundant bacterial phyla in mucosal scrapings and faecal content. **A** Sample from the non-infected groups (bLF and PBS), **C** Samples from pigs challenged with STEC (STEC and STEC + bLF). **B** Relative abundance (RA) microbiota mucosal scrapings Ileum from the non-infected group, **D** RA microbiota mucosal scrapings ileum from the STEC challenge group. bLF: bovine lactoferrin, LP: lamina propria, PP: Peyer’s patches, STEC: Shiga toxin-producing *E. coli*. Data was shown as mean ± SD; **P < 0.01

To examine the impact of bLF on the gut microbiota in more detail, we compared the relative abundance (RA) of individual phyla between the treatment groups. This revealed that the Firmicutes phylum was the most dominant phylum in all tested samples (Fig. 4A and C). In the STEC challenge experiment, administration of bLF significantly increased the RA of Actinobacteria in both the ileal mucosal scrapings with PP (*P* = 0.0079) and without PP (*P* = 0.0079) (Fig. 4D). A similar observation was found for the samples of the microbiome trial, where bLF administration tended to increase the RA of Actinobacteria in the ileal mucosal scrapings with PP (*P* = 0.1037) (Fig. 4C). BLF did not significantly impact the phylum-level RA of the faecal swab samples in both the microbiome and STEC challenge trials.

**Fig. 5:**
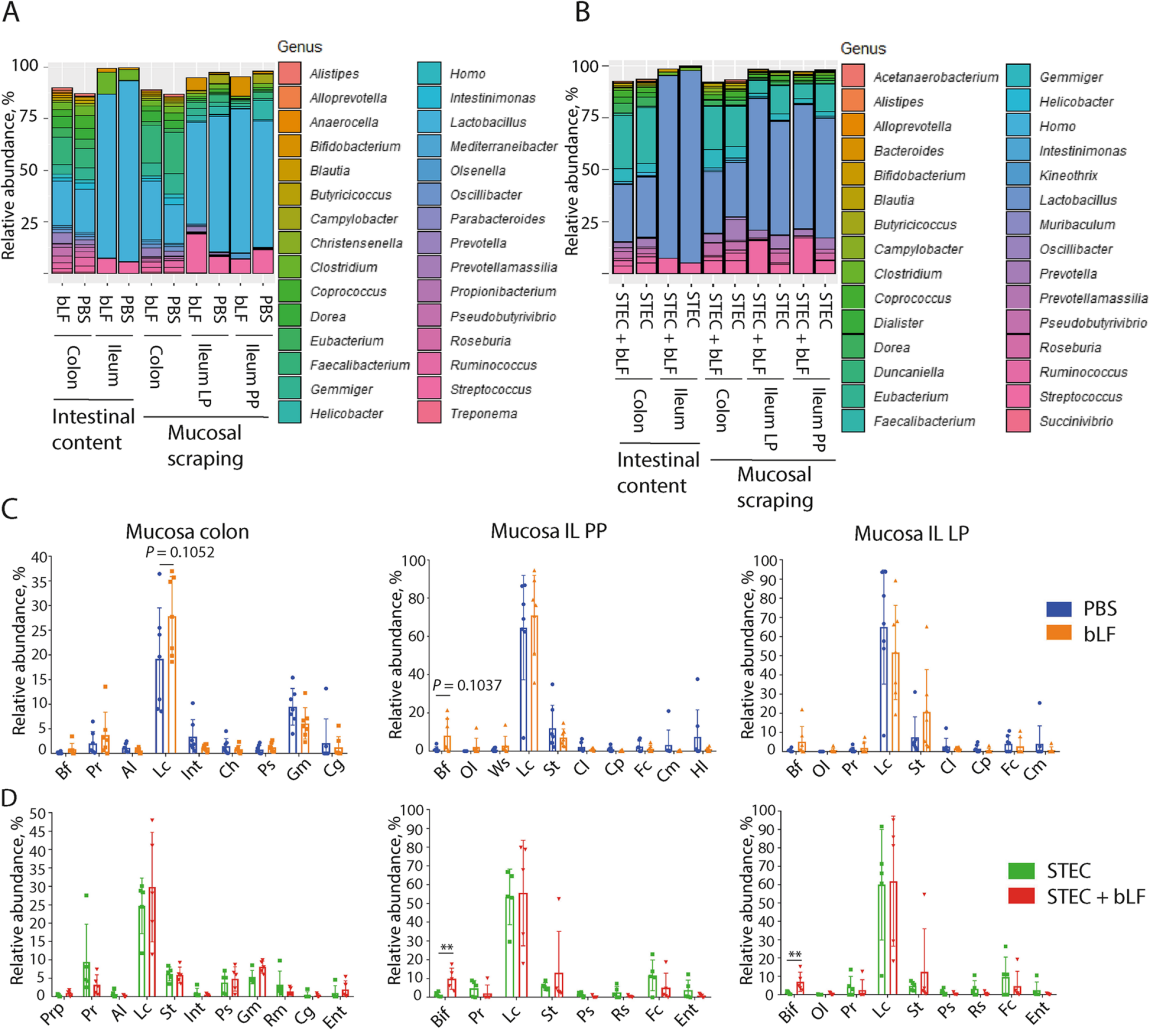
Effects of LF administration on the relative abundance of bacterial genera. **A** and **B** Stacked bar charts of the 30 most abundant bacterial genera identified in the faecal content and mucosal scrapings in unchallenged piglets **(A)** and in F18^+^ STEC challenged piglets **(B)**. **C** and **D** Relative abundance of bacterial genera in the mucosal scrapings of colon and ileum with PP and without PP (=ileal LP) in unchallenged piglets **(C)** and in F18^+^ STEC challenged piglets **(D)**. Al: *Alistipes*, Bf: *Bifidobacterium*, Bif: Bifidobacteriaceae (f), Cg: *Candidatus* Glomeribacter, Ch: *Christensenella*, Cl: *Clostridium*, Cm: *Campylobacter*, Cp: *Coprococcus*, Ent: Enterobacteriaceae (f), Fc: *Faecalibacterium*, Gm: *Gemmiger*, Hl: *Helicobacter*, Int: *Intestinimonas*, Lc: *Lactobacillus*, Ol: *Olsenella*, Pr: *Prevotella*, Prp: *Propionibacterium*, Ps: *Pseudobutyrivibrio*, Rm: *Ruminococcus*, Rs: *Roseburia*, St: *Streptococcus*, Ws: *Weissella*, bLF: bovine lactoferrin, IL: ileum, LP: Lamina propria, PP: Peyer’s patches, STEC: Shiga toxin-producing *E. coli*. Data was shown as mean ± SD; **P < 0.01

We also assessed the impact of bLF on individual genera in weaned pigs, under both STEC challenge and unchallenged conditions, as LF is frequently associated with increased RA of *Lactobacillus* and *Bifidobacterium* genera [28, 46, 47]. When studying RA of the 30 most abundant genera, *Lactobacillus* was found to be the most abundant genus in the intestinal content and mucosal scrapings (Fig. 5A and B). However, in this study, bLF did not increase the RA of the *Lactobacillus* genus in any of the samples (Fig. 5C and D). On the other hand, in mucosal scrapings of ileum with PP and without PP (= ileal LP), bLF treatment significantly increased the RA of *Bifidobacterium* in challenged piglets (*P* = 0.0079; Fig. 5C and D), while the RA of *Bifidobacterium* in unchallenged piglets tended to increase in the mucosal scrapings of ileal PP (*P* = 0.1037; Fig. 5C and D). In both the microbiome and STEC challenge trials, bLF did not exhibit a significant impact on the genus-level composition of the microbiota in the faecal swab samples.

### Effect of lactoferrin on the faecal shedding of F18^+^ STEC in weaned piglets

In addition to examining the effect on the microbiome, we aimed to evaluate the ability of bLF to prevent F18^+^ STEC infections by investigating faecal excretion of the challenge strain and the induced immune responses. Upon euthanasia, we assessed the F18 receptor status through an in vitro villus adhesion assay, which revealed that 2 out of 5 piglets in both the STEC and STEC + bLF groups were F18 receptor negative. Consequently, these piglets were excluded from the analyses studying the impact of bLF on the faecal shedding of the F18^+^ STEC strain and F18-specific immune responses, as these piglets were not susceptible to an F18^+^ STEC infection.

**Fig. 6:**
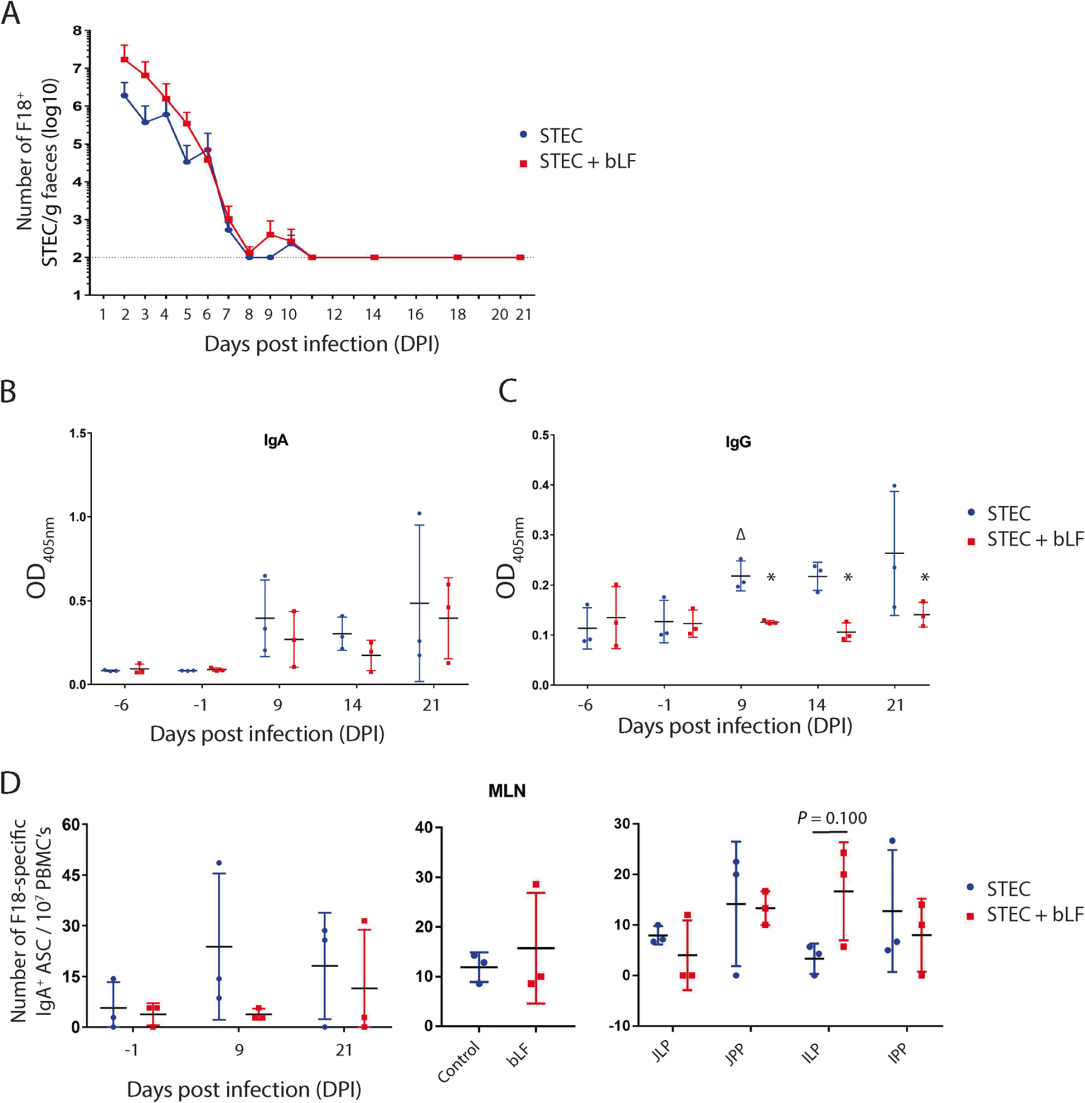
Effect of bovine lactoferrin on the F18^+^ STEC excretion and immune modulatory effect upon F18^+^ STEC challenge infection. **A** Mean faecal excretion of F18^+^ STEC (log10)/g faeces (± SD). The mean faecal excretion of F18^+^ STEC was calculated by plating faecal dilutions and confirmed by dot blot analysis. The detection limit is represented by a dotted line at 2 log_10_ STEC/g faeces. **B** and **C:** Effect of bovine lactoferrin on the F18-specific serum antibody levels upon F18^+^ STEC challenge, **B** F18-specific IgA and **C** IgG serum responses. **D** F18-specific IgA^+^ antibody secreting cells after oral administration of bovine lactoferrin. ELISpot of F18-specific IgA^+^ ASCs from PBMCs (left) isolated on −1, 9 and 21 days post infection (dpi) and mononuclear cells isolated from mesenteric lymph nodes (middle) and intestinal tissues (right) at 21 dpi. ASC: antibody secreting cells, bLF: bovine lactoferrin, ILP: ileal lamina propria, IPP: ileal Peyer’s patches, JLP: jejunal lamina propria, JPP: jejunal Peyer’s patches, MLN: mesenteric lymph nodes, PBMC: peripheral blood mononuclear cell. Data was shown as mean ± SD; ^*^Q < 0.05; ∆ *Q* < 0.05 (*n* = 3/group)

In order to evaluate the effect of bLF on shedding of the F18^+^ STEC strain, the F18^+^ STEC in faecal samples were enumerated. Both challenge groups, with or without bLF administration, displayed a high level of shedding on D2 post-infection (6.28 log_10_ CFU F18^+^ STEC/g faeces in the control group versus 7.23 log_10_ CFU F18^+^ STEC/g faeces in the bLF group). Over the following 4 d, excretion levels declined but remained elevated (approximately 4.50 to 6.50 log_10_ CFU F18^+^ STEC/g faeces) in both groups (Fig. 6A). From D6 to D8 post-infection, the excretion of F18^+^ STEC strain decreased rapidly, but faecal excretion rose again from D8 to D11 post-infection. Nonetheless, from D11 post-infection onwards, the faecal shedding of F18^+^ STEC remained below the detection threshold (Fig. 6A). In conclusion, bLF did not reduce faecal excretion of the F18^+^ STEC strain in this experimental setup.

### Impact of bLF on F18-specific immune responses

Given the immunomodulatory nature of bLF, we also investigated whether bLF could impact the F18-specific immune responses following infection. To this end, serum F18-specific IgG and IgA responses were evaluated on D−6, D−1, D9, D14, and D21.

Prior to the challenge infection (D−6 and D−1), there was little to no detection of F18-specific IgA and IgG serum responses, indicating that there was no previous exposure to the pathogen (Fig. 6B–C). F18-specific IgA antibodies were detected in serum on D9 post-infection and remained elevated at D14 and D21 post-infection in both the STEC and STEC + bLF group, but the increase was not significant compared to D−1. In contrast, the F18-specific IgG serum levels showed a significant increase in the STEC group at D9 post-infection compared to the pre-infection values (D−1, *Q* = 0.0084, indicated as ∆ in Fig. 6C) and remained elevated at D14 and D21 post-infection. However, in the STEC + bLF group, no significant increase in the F18-specific IgG serum response was observed following challenge infection compared to pre-infection values. As compared to the STEC group, STEC + bLF significantly decreased the F18-specific IgG serum response on D9, D14, and D21 post-infection (*Q* = 0.0409, *Q* = 0.0231, and *Q* = 0.0231, respectively; indicated as * in Fig. 6C).

To further evaluate the effect of bLF on the intestinal immune response against F18-fimbriated STEC, we determined the number of circulating F18 fimbriae-specific IgA^+^ ASCs as well as those residing in different gut tissues at D21 post infection by ELISpot. As shown in Fig. 6D, circulating F18 fimbriae-specific IgA^+^ ASCs were detected at D−1, D9 and D21, but no significant increase in F18-specific IgA^+^ ASCs could be observed in either groups following challenge infection. Likewise, no significant differences were observed in the number of F18-specific IgA^+^ ASCs between both groups in all tissues (Fig. 6D). However, the number of F18-specific IgA^+^ ASC in the Ileal lamina propria (ILP) tended to increase upon administration of bLF (*P* = 0.1000; Fig. 6D).

## Discussion

The use of antibiotics has reduced morbidity and mortality associated with bacterial infections. However, their extensive and sometimes inappropriate use in food producing animals has led to the rapid spread of antibiotic resistance, causing major health risks in both animals and humans [48–50]. Considering the projected increase in global demand for animal protein, alternatives will be crucial in combatting antimicrobial resistance [51]. LF is one of these potential alternatives as it has demonstrated broad-spectrum antibacterial activity against Gram-positive and Gram-negative bacteria, both in vitro and in vivo [52]. Our previous studies have clearly demonstrated LF's capability to inhibit bacterial growth, degrade ETEC/STEC associated virulence factors and attenuate ETEC-induced fluid secretion [29, 30]. Moreover, LF has been recognized for its impact on the bacterial composition within the gastrointestinal tract. The latter is colonised by numerous bacteria creating an intricate relationship between the gut microbiome and its host [53]. More specifically, the gut microbiome aids in nutrient absorption, regulates the intestinal barrier, plays a role in the metabolism of bile acids and influences immune function [54, 55]. Therefore, we wanted to assess the effect of bLF on the microbiome of healthy and STEC challenged piglets. In our study, the oral administration of bLF had no effect on the bacterial richness and diversity indices. This contrasts previous results in suckling piglets, in which the ACE and Chao1 indices were increased in the bLF group [26]. The observed differences could potentially be attributed to the higher bLF concentration (500 mg/kg/d) compared to 500 mg/piglet/d in this study. Moreover, both the iron saturation level of bLF and the gender of the piglets, used in the abovementioned study, can be potential sources of variation but were not specified. Furthermore, the inclusion of sow milk during the suckling period further increases LF intake and may have contributed to the observed differences [26]. On the other hand, the observed differences may also be in part due to rapid transformation of the microbiome of suckling piglets. This transformation is characterized by an increase in species richness as the piglets age [56]. In this context, the supplementation of LF during the suckling period may assist in expediting the development towards a rich and diverse microbial community upon weaning. This high microbial diversity corresponds to a more developed gut ecosystem and supports the concept of functional redundancy. This concept suggests that the presence of additional taxa adds redundancy, enhancing the ecosystem's ability to maintain resilience and stability [56, 57]. Furthermore, alongside the rise in α-diversity during early life, multiple studies have documented a decrease in the variability of β-diversity, as piglets mature. This finding indicates a convergence towards a homogeneous, diverse, and stable microbial composition following weaning [58, 59]. The latter, in combination with the lower bLF concentration used in this study, could potentially explain why LF did not significantly impact the β-diversity of weaned piglets, as previously observed in 21 days old pre-weaned piglets [26].

Additionally, our study shows that oral administration of bLF resulted in an increased RA of both *Bifidobacterium* and its corresponding phylum, Actinobacteria, in the ileal mucosa. These Actinobacteria, one of the four major phyla within the gut microbiota, play a vital role in maintaining gut homeostasis [60]. Over the past decade, there has been a growing focus on Actinobacteria, particularly their contributions to both gastrointestinal and systemic diseases and their potential therapeutic applications. Notably, certain classes within this phylum, such as *Bifidobacterium*, have gained significant attention as probiotics due to their demonstrated positive effects in various pathological conditions [60–62]. One key mechanism through which Actinobacteria, including *Bifidobacterium*, exert their beneficial influence is by generating short-chain fatty acids (SCFA) like acetate, propionate, and butyrate through the fermentation of carbohydrates. These SCFAs serve multiple crucial functions, including providing energy for the turnover of epithelial cells and exhibiting potent antibacterial properties [63–65]. Our observations regarding the increased RA of *Bifidobacterium* align with previous findings. For example, in pigs, the administration of human LF (hLF) and bLF, can promote the growth of beneficial microbes, such as *Bifidobacterium *spp. and *Lactobacillus* spp., while reducing the growth of Gram-positive and Gram-negative pathogenic microbes, such as *Salmonella* and *Staphylococcus aureus* [26, 28, 66]. Similar observations were found for human infants, revealing a positive correlation between the concentration of hLF in faeces and the levels of faecal Bifidobacteria and Lactobacilli [27]. Interestingly, recombinant lactoferrampin-lactoferricin fusion constructs, produced by *Pichia pastoris* or *Photorhabdus luminescens*, also increased the amount of Lactobacilli and Bifidobacteria in the ileum and colon of weaned pigs [67, 68]. Overall, these studies show the potential of LF to modulate the microbial ecosystem, promoting a healthier microbiota composition and supporting overall gut health in both animals and humans.

While most *E. coli* strains are beneficial, aiding in digestion and producing vitamins, certain strains can cause illness, particularly diarrhoea [69]. F18ab- and F18ac-fimbriated *E. coli* strains are commonly associated with oedema disease and PWD, respectively. These strains are responsible for considerable financial losses caused by an increased mortality, reduced weight gain and expenses incurred for treatments, such vaccinations, antibiotic use and feed supplements [1, 70]. The emergence of *E. coli* strains isolated from pigs carrying colistin resistance genes on transferable plasmids is concerning [5, 71]. This is due to the use of colistin as a last-resort antibiotic in the treatment of multidrug-resistant bacteria in humans, while also posing a challenge in the treatment of PWD [9]. Our previous studies support LF's potential as an alternative strategy to prevent *E. coli* infections in pigs, by reducing the adhesion of ETEC strains to intestinal epithelial cells, in vitro and in vivo, while ameliorating the ETEC-induced fluid loss [29, 30]. Here, we wanted to further evaluate the in vivo effect of bLF by assessing its effect on bacterial excretion and modulation of the immune response upon an experimental infection. The present study demonstrated that bLF had no significant effect on the excretion of an F18-fimbriated *E. coli* strain, which is in line with previous findings using hLF [72, 73]. Furthermore, several other studies have reported that LF can decrease the occurrence of diarrhoea, while improving growth performance [74, 75]. However, in this study, we could not assess these parameters as the F107/86 strain is used as a colonization model rather than a disease model, primarily due to its inability to produce LT and ST enterotoxins. Furthermore, the number of animals used is not sufficient to adequately assess the impact on growth performance. In another study, a genetically modified *Lactobacillus plantarum* strain was created that produces pLF. Incorporating this modified strain into the diet led to a significant increase in average daily weight gain and reduced the occurrence of diarrhoea [76]. In addition, a genetically modified *Lactobacillus reuteri* strain encoding for a lactoferricin-lactoferrampin fusion construct (LR-LFCA), revealed that oral administration of LR-LFCA to neonatal piglets effectively reduced F4^+^ ETEC induced weight loss and diarrhoea. Moreover, the same study also demonstrated significantly elevated serum IgG levels and IgA in the mucosa of the jejunum and ileum, compared to the ETEC LR-control group [77]. In contrast, the administration of bLF in this study tended to increase the number of F18-specific IgA^+^ ASC, but only in the ileal mucosal tissue without PP. Furthermore, F18-specific IgG serum levels were not increased in the bLF group at D9, D14 and D21 post infection. In fact, at these timepoints, the F18-specific IgG levels were significantly reduced compared to the infection control group. We speculate that the lack of an F18-specific IgG response could be attributed to the proteolytic activity of bLF [30]. The latter could reduce the exposure of the immune system to the F18^+^ challenge strain by decreasing the adherence of the F18^+^ *E. coli* strain to the intestinal epithelial cells [29]. Previously, a similar immune-exclusion phenomenon was observed in pigs receiving feed containing mVHH-IgA targeting F4^+^ ETEC. The latter led to a reduced seroconversion against F4^+^ ETEC upon an experimental challenge infection [78]. However, a similar reduction in serum IgA levels would be expected upon a reduce exposure to the F18 STEC strain, which was not observed here. It is therefore possible that another mechanism is responsible for the absence of a serum IgG response. Further research is necessary to determine the cause behind the IgG immune exclusion phenomenon upon administration of bLF.

Since the influence of LF extends beyond its direct impact on the pathogen, it is plausible that an alternative mechanism is responsible for the absence of a serum IgG response. Therefore, exploring the intricate interplay of different LF variants, including pLF, in the context of *E. coli* infections in piglets could provide a more comprehensive understanding of their role in modulating the immune response in piglets during *E. coli* infections.

## Conclusion

In summary, the oral administration of bLF did not have a notable impact on the α- and β-diversity of the gut microbiome. However, it did increase the RA of the Actinobacteria phylum and the *Bifidobacterium* genus, which play an important role in maintaining gut health. The reasons for LF's ability to inhibit the induction of F18-specific IgG responses remain unknown. Interestingly, this phenomenon does not apply to IgA, suggesting that it may be more related to an immune-modulating effect of LF.

## Data Availability

All sequencing data are deposited at NCBI Short Reads Archive (SRA), project accession number PRJNA1008698.

## Abbreviations

ABTS: 2,2'-azino-bis (3-ethylbenzothiazoline-6-sulfonic acid)
AEC: 3-amino-9-ethylcarbazole
ASC: Antibody secreting cell
bLF: Bovine lactoferrin
DPI: Days post infection
*E. coli*: *Escherichia coli*
EHEC: Enterohemorrhagic *E. coli*
ELISpot: Enzyme-linked immunosorbent spot
ETEC: Enterotoxigenic *E. coli*
hLF: Human lactoferrin
IL: Ileum
ILP: Ileal lamina propria
IPP: Ileal Peyer’s patches
JLP: Jejunal lamina propria
JPP: Jejunal Peyer’s patches
LF: Lactoferrin
LP: Lamina propria
LPS: Lipopolysaccharides
LR-LFCA: *Lactobacillus reuteri* encoding for a lactoferricin-lactoferrampin fusion construct
LT: Heat-labile enterotoxin
MAMP: Microbe-associated molecular patterns
MED: Minimum entropy decomposition
MC: Mononuclear cell
MLN: Mesenteric lymph nodes
OTU: Operational taxonomic unit
PBMC: Peripheral blood mononuclear cell
PCoA: Principal coordinates analysis
PERMANOVA: Permutational multivariate analysis of variance
pLF: Porcine lactoferrin
PP: Peyer’s patches
PWD: Post-weaning diarrhoea
RA: Relative abundance
SCFA: Short-chain fatty acids
ST: Heat-stable enterotoxin
STEC: Shiga toxin-producing *E. coli*
Stx2e: Shiga toxin type 2e
TMB: 3,3′,5,5′-Tetramethylbenzidine
UP: Ultrapure water
